# Union of the American and the Southern Dental Associations

**Published:** 1897-10

**Authors:** J. M. Walker


ARTICLE III.

### UNION OF THE AMERICAN AND THE SOUTHERN DENTAL ASSOCIATIONS.

**Organization of the National Dental Association.**
**The American Dental Association adjours *sine die.***
**The Southern Dental Association Re-Organizes as The Southern Branch of the National Dental Association, to be known as The Southern Dental Association.**

HYGEIA HOTEL,
OLD POINT COMFORT, VIRGINIA,
AUGUST, 1897.

The Southern Dental Association was called to order for its twenty-eighth annual meeting, at 11 a. m., Tuesday, August 3, 1897. The president Dr. William H. Richards, of Knoxville, Tenn., in the chair. The American Denta

Association met at the Chamberlain Hotel at the same hour, the president, Dr. James Truman, Philadelphia; in the chair. Mutual courtesies of invitation, etc., were tendered and formally adopted.

In both associations the report of their respective Committees on Union was made the special order of business for Wednesday afternoon session, at which time Dr. L. G. Noel, chairman of the committee from the Southern Dental Association, read the following report:

Your committee has considered the subject of Union of the Southern Dental Association and the American Dental Association, and beg leave to make the following report:

The Union of the two associations seems to them a desirable object to be attained, and they, in conference with the committee from the other association, have devised a plan, making the following provisions:

*First:* A new name.

*Second:* Assuring the membership in the new association of all members of the Southern Dental Association and the American Dental Association.

*Third:* Providing for the organization of branches in the several divisions of the country, one of which shall be a continuation of the Southern Dental Association in the South, thus preserving in its entirety, this organization to us, this organization which has become so dear to our hearts during all the years of its struggles and success. Its history and autonomy will be thus preserved.

*Fourth:* The plan contemplates dividing the country into three parts (or divisions) and holding the annual meetings by rotation in the several divisions; thus, in 1898 the meeting will be held in the West, in 1899 in the East.

*Fifth:* The plan we would suggest also provides for the selection of affairs in like manner, by rotation from the several divisions of the country.

Your committee would respectfully suggest that an early time be fixed for holding a general conference of the members of the two associations, with a view to perfecting a plan of union.

(Signed) L. G. NOEL,
E. P. BEADLES,
GEO. EUBANK,
J. ROLLO KNAPP,
V. E. TURNER.

Printed copies of a proposed constitution for the new organization had been mailed to all the members of the two associations previous to the meetings but numerous alterations and amendments having been subsequently made by the joint committee, Dr. Noel proceeded to read the proposed preamble, constitution, rules of order, order of business, and standing resolutions as amended up to the hour of reading.

At the conclusion of the reading Dr. J. Rollo Knapp, New Orleans, offered a motion to the effect: "It is the sense of this society that the report of this committee be accepted, and that the union be effected."

On motion, Dr. Fillebrown, Boston, chairman of the committee of the American Association, spoke at some length on the many advantages offered by the proposed consolidation of the two associations.

After some discussion Dr. Knapp's motion was put to vote and passed by a decided majority.

On motion of Dr. Cowardin, of Richmond, the same committee was continued to confer with the other committee and fix upon a time for a joint meeting of both associations for the purpose of effecting the union. Similar action was taken in the American Association and a joint session of the two associations was accordingly held in the spacious ball-room of the Hygeia Hotel, at 12.30, on Thursday, Aug. 5.

The meeting was called to order by Dr. Thomas Fillebrown, of Boston, chairman of the joint committee on consolidation.

On motion, a temporary organization was effected by the election of Dr. John B. Rich, of Washington, D. C., one of the oldest practicing dentists in the United States, as temporary chairman. Dr. W. E Walker, of Pass Christian, Miss., was elected secretary *pro tem.*

Dr. George H. Cushing, of Chicago, was selected corresponding secretary *pro tem.*

Dr. Patterson offered the following resolution, which was adopted without discussion :

*Resolved:* That the members of the American Dental Association and the members of the Southern Dental Association do hereby organize themselves into a body, to be known and styled as "The National Dental Association."

Dr. John S. Marshall offered the following :

*Resolved,* That the constitution, by-laws and rules of order decided upon and presented by the joint committee of the two bodies be and are hereby adopted as the constitution, by-laws and rules of order of "The National Dental Association." Adopted.

A motion was offered by Dr. Taft that the secretary read the constitution, by-laws and rules of order, in order that the members may better understand it.

The motion was carried and the secretary read the constitution, rules of order, standing resolutions, etc., as adopted.

The next business before the meeting being the election of permanent officers, Drs. Taft and Nobles were appointed tellers and an informal ballot taken.

Drs. J. B. Rich, James McManus, W. W. H. Thackston, Truman, Brophy, Taft, Peirce, Beach, Crouse, B. Holly Smith and H. A. Smith received complimentary votes, Dr. Thomas Fillebrown, Boston, receiving nearly two-thirds of all the votes cast. On motion of Dr. J. Hall Moore, the informal ballot was made formal, and on motion of Dr. Beach, the rules were suspended and the secretary cast the unanimous vote of the National Dental Association for Dr. Thomas Fillebrown as its first presieat. (Great Applause.) The temporary chairman then appointed Dr. James Truman, of Philadelphia, and Dr. W. H. Richards, of Knoxville, Tenn., presidents respectively of the American and Southern Associations to escort the president, Dr. Fillebrown, to the chair.

These gentlemen presented the new president to the convention.

Dr. Fillebrown accepted the honor bestowed upon him in a graceful speech. He said that he had witnessed to-day what he had desired and labored for over twenty years, the organization of a broad national association, and he could say, like the prophet of old, "Now, Lord, let Thy servant depart in peace, for mine eyes have seen Thy consolation." He was heartily applauded.

On motion of Dr. Beach, of Virginia, the convention then adjourned to 4.30 o'clock p. m.

AFTERNOON SESSION.

On motion of Dr. Crawford, by unanimous consent, the office of assistant secretary was created, and made a matter of record.

Dr. Geo. H. Cushing, of Chicago, was elected secretary, and on motion of Dr. C. N. Peirce, he was authorized to cast the ballot for Dr. W. E. Walker, of Pass Christian.

The following officers were then elected :

Vice-President from the Eastern division, Dr. Jas McManus, of Hartford, Conn.

Vice-President from the Western division, Dr. L. L. Dunbar, of San Francisco, Cal.

Vice President from the Southern division, Dr. B. Holly Smith, of Baltimore.

Corresponding Secretary, Mrs. Emma Eames Chase, of St. Louis.

Treasurer, Dr. Henry W. Morgan, of Nashville, Tenn.

Executive Committee.—The chair announced that the executive committee was composed of nine members, three of whom are elected at their first election for three years, three for two years and three for one year, and that one of each three must be selected from each division. Before the convention proceeded to an election, Dr. E. P. Beadles offered the following :

*Resolved*, That the president cast the ballot for the executive committee for members of the association best suited for the position.

The resolution was adopted.

The president subsequently announced the following as the names of the committee :

To serve three years—Dr. J. D. Noel, Dr. J. N. Crouse, Dr. V. H. Jackson.

To serve two years—Dr. M. F. Finley, Dr. J. D. Patterson, Dr. H. A. Smith.

To serve one year—Dr. Geo. Eubank, Dr. G. V. I. Brown, Dr. C. N. Peirce.

Dr. John S. Marshall offered a resolution that the treasurers of the American and of the Southern Dental Association furnish the treasurer of the National Dental Association with the names of all the members of the associations in good standing at the time of adjournment, and that he be directed to add their names to the constitution as permanent members and that this act be as legal and binding as if the signatures had been affixed by the individuals themselves.

The motion was adopted.

Omaha, Minneapolis and Denver were put in nomination as the next place of meeting of the convention. A ballot was taken and Omaha receiving the majority of the votes cast was announced as the next place of meeting of the association.

On motion of Dr. John S. Marshall, the president was requested to appoint a committee of three to take under consideration the establishment of a journal for publishing the annual proceedings of the convention, with other original matter pertaining to surgery and the collateral sciences. and to report at the next meeting.

The following offered by Dr. Crawford was adopted :

*Resolved*, That it is the sense of the National Dental Association that any member of the dental profession in good standing presenting a certificate of registration from any State in the Union, that the same should be admitted to registration in any other State of the Union, when presenting such certificates of registration and good standing professionally, without an additional examination.

On motion, the president was instructed to have a sufficient number of corrected copies of the constitution and by-laws printed for the use of the members of the association.

To meet this and other expenses of the National Association, who has an empty treasury, the dues of present members having been paid to the American and the Southern Associations respectively; the treasurers of the two associations were attributed to turn over to the treasurer of the National Association one dollar for each member in good standing.

On motion of Dr. R. Finley Hunt, the president was authorized to appoint a committee looking to the preparation of a history of the dental profession in the United States.

Dr. Crouse offered a resolution to the effect that any member of the dental profession who has been a reputable practitioner for fifty years may be elected a permanent member of the National Dental Association without payment of dues.

This was unanimously adopted.

On motion of Dr. M. F. Finley, a motion in the interests of public health was adopted looking to the employment by the army medical museum and library of a dentist to be selected for his eminent ability and fitness, whose time shall be wholly devoted to the advancement of dental science.

On motion, a committee of three—Drs. Taft, Crawford and McManus—was appointed to confer with state and local societies to aid in the formation of new societies, etc.

On motion, it was resolved, that the proceedings of the present meeting of the National Dental Association be attached as supplementary to the proceedings of both the American and the Southern Dental Association and incorporated in their published reports.

On motion of Dr. Richards, the two associations were requested to deposit their gavels in the Army Medical Museum at Washington.

A motion by Dr. J. Y. Crawford, that the name of the new organization be changed to that of "The American Association of Dental Surgeons," caused an animated debate and brought out the suggestion of other names—Association of Stomatologists; Association for the advancement of Dental Science, etc., etc. There being objections made to all the proposed changes they were laid over for consideration at the next annual meeting, and the association adjourned to meet at Omaha in 1898.—MRS. J. M. WALKER for *Ohio Dental Journal.*

